# Association Between Inflammation and Risk of Cardiovascular Mortality in Cancer Survivors

**DOI:** 10.1016/j.jacadv.2025.102565

**Published:** 2026-01-23

**Authors:** Quan Yang, Jiazhen Zheng, Chunting Zhao, Min Wu, Run Wang, Mingya Liu, Kai-Hang Yiu

**Affiliations:** a Cardiac and Vascular Center, Division of Cardiovascular Medicine, The University of Hong Kong-Shenzhen Hospital, Shenzhen, China; b Shenzhen Clinical Research Center for Rare Diseases, Shenzhen, China; c Bioscience and Biomedical Engineering Thrust, Systems Hub, The Hong Kong University of Science and Technology (Guangzhou), Guangzhou, Guangdong, China; d Cardiology Division, Department of Medicine, The University of Hong Kong, Queen Mary Hospital, Hong Kong, China

**Keywords:** cancer, cardio-oncology, cancer survivors, mortality, omega-3 supplement

## Abstract

**Background:**

Cancer and cardiovascular disease are closely linked, contributing to high mortality rates and significant disease burden. Inflammation serves as a common risk factor for both conditions, yet there is a lack of data on systemic inflammation and its impact on cardiovascular mortality in cancer survivors.

**Objectives:**

This study aimed to explore the relationship between inflammation and cardiovascular mortality in cancer survivors.

**Methods:**

We analyzed 15,420 UK Biobank participants with prior cancer and no active therapy. Multivariable analyses using competing-risk models were conducted to assess the association between C-reactive protein (CRP) levels and the risk of cardiovascular mortality in cancer survivors.

**Results:**

During a median follow-up of 10.2 ± 1.9 years, there were 1,167 deaths among cancer survivors (7.6%), including 813 cancer-related deaths and 103 cardiovascular deaths. Unadjusted competing-risk analysis revealed that CRP levels >2 mg/L conferred a 1.12-fold higher risk of cardiovascular death in cancer survivors compared to CRP ≤2 mg/L (sub-distribution hazard ratio [sHR]: 2.12; 95% CI: 1.40-3.21; *P* < 0.001). After adjusting for sociodemographic, lifestyle factors, and clinical characteristics, CRP levels >2 mg/L remained linked to a 64% increased risk of cardiovascular death (sHR: 1.64; 95% CI: 1.07-2.50; *P* = 0.023). Subgroup analyses further confirmed the consistent finding.

**Conclusions:**

This study demonstrates that chronic inflammation is significantly associated with cardiovascular mortality in cancer survivors. Future research should focus on whether targeting inflammatory pathways can reduce the burden of cardiovascular mortality in this population.

Cancer and cardiovascular disease (CVD) have become the leading causes of death from noncommunicable diseases globally.^1^ Advances in cancer treatments have significantly improved survival rates, but these treatments may have cancer therapy-related cardiovascular toxicity that is increasingly recognized.^2^ Some research suggests that the risk of cardiovascular death in cancer survivors may eventually surpass the risk of death from cancer itself.^3^

C-reactive protein (CRP), an inflammatory marker, has been extensively studied in the context of both cancer and CVD. Elevated CRP levels have been linked to all-cause mortality and cancer-related deaths in cancer patients,^4^, ^5^, ^6^, ^7^ as well as to cardiovascular deaths in the general population,^8^ healthy women,^9^ and individuals with CVD.^10^^,^^11^ Anti-inflammatory treatments have been shown to reduce both cardiovascular events and cancer occurrences.^12^ However, there is a gap in research regarding the relationship between CRP levels and cardiovascular deaths specifically in various types of cancer survivors.

The aim of this study was to investigate the relationship between CRP levels and cardiovascular death in various types of cancer survivors using data from the widely validated UK Biobank.^13^ By examining this relationship, we hope to enhance understanding of cardiovascular death incidence among cancer survivors and explore the potential of CRP as a biomarker for risk assessment in this population. Future research should assess whether targeted anti-inflammatory therapies could reduce cardiovascular death in cancer survivors, potentially bridging the gap between oncology and cardiology care.

## Methods

### Study population

The UK Biobank includes over 500,000 participants aged 40 to 69 years from across the United Kingdom. Baseline assessments gather demographic information, detailed lifestyle and medical characteristics, and a range of physical measurements and blood samples. The UK Biobank protocol is publicly accessible. Health outcomes are linked to both Hospital Episode Statistics and death registers, with data recorded using standardized International Classification of Diseases codes.

**Figure 1:**
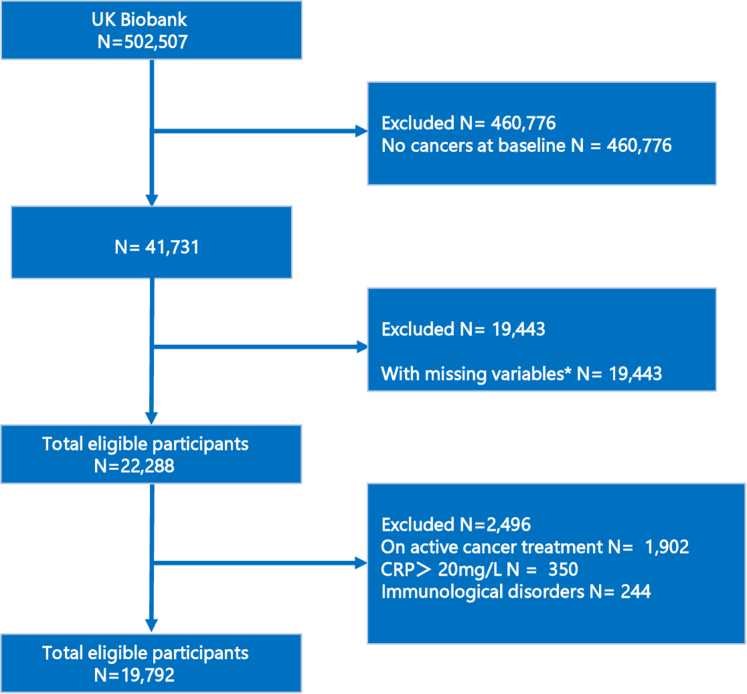
**Flow Diagram of the Study Population** ∗Missing variables include age, sex, ethnic, education, body mass index, waist circumference, number of times per week consuming processed meats, number of times per day consuming fruit or vegetables, smoking history, alcohol consumption, physical activity, C-reactive protein, low-density lipoprotein cholesterol, high-density lipoprotein cholesterol, triglyceride, family history of cardiovascular disease, antihypertensive medication, diabetes medication, and statin. CRP = C-reactive protein.

At baseline, there were 41,731 cancer survivors in the UK Biobank. We sequentially excluded 19,443 individuals with incomplete baseline data, 1,902 who received radiotherapy or chemotherapy after enrollment, 350 with evidence of acute infection (CRP >20 mg/L),^14^ and 350 with a documented immunological disorders. These criteria yielded an analytic cohort of 19,792 cancer survivors. The exact distribution of missing values is itemized as follows: ethnicity, meat and vegetable consumption, alcohol intake, and physical activity, 29; education, 305; body mass index (BMI), 178; waist circumference, 112; total cholesterol (TC), 3,157; low-density lipoprotein cholesterol (LDL-C), 3,229; high-density lipoprotein cholesterol (HDL-C), 6,477; triglycerides (TG), 3,191; family history of CVD, 3,944; and medication use, 7,819 (Figure 1). Of 19,792 cancer survivors, 7,710 presented with CRP >2 mg/L. A 1:1 propensity score matching cohort was constructed by pairing these individuals to survivors with CRP ≤2 mg/L on age and sex, yielding a standardized mean difference <0.1 and a final analytic sample of 15,420 patients. The UK Biobank study was approved by the North West Multicenter Research Ethics Committee. All participants provided informed consent during their initial visit to the assessment center. The UK Biobank project number for this study is 97089.

### Covariables and outcomes

By reviewing existing guidelines,^2^ we identified potential determinants of CRP levels and cardiovascular mortality in cancer survivors. From the baseline assessment data, we extracted the following variables: sociodemographic factors (eg, age, sex, ethnicity, education level); lifestyle characteristics (eg, BMI, waist circumference, smoking history, alcohol consumption, dietary intake, physical activity); and clinical characteristics (eg, TC, LDL-C, HDL-C, TG, family history of CVD, diagnoses of diabetes, hypertension, hypercholesterolemia, coronary artery disease (CAD), atrial fibrillation, ischemic stroke, heart failure, chemotherapy, radiotherapy and use of antihypertensive medications, statins, or diabetes medications). Proportionality of hazards was verified for all covariates in both competing-risk and Cox models (global test *P* > 0.05 for each), and multicollinearity was excluded (variance inflation factor <7 for every variable). We tested for biological interaction between CRP and age, sex, BMI, smoking status, TG, history of hypertension, diabetes, and CAD; false-discovery-rate-adjusted *P* values exceeded 0.10 for every comparison, indicating no statistically significant effect modification.

In cancer survivors who had completed chemotherapy and/or radiotherapy, baseline CRP was quantified by high-sensitivity immunoturbidimetry on the Beckman Coulter AU5800 platform. The UK Biobank’s standardized protocol incorporated rigorous quality-control checkpoints spanning sample collection, processing, recovery, and real-time assay performance monitoring. Consistent with established thresholds for residual inflammatory risk, CRP was dichotomized at 2 mg/L.^11^^,^^15^^,^^16^

We stratified variables such as age (using a threshold of 65 years), BMI (using a threshold of 30 kg/m^2^), waist circumference (using a threshold of 90 cm), LDL-C (according to guideline recommendations), history of hypercholesterolemia (according to guideline recommendations), and HDL-C and TG (according to guideline recommendations).^17^ All-cause, cardiovascular, and cancer mortality outcomes were determined using death registry data. The mean follow-up period for Hospital Episode Statistics and mortality data was 10.2 ± 1.9 years (IQR: 9.8-11.3 years). Further details on the covariates, how they were measured, and the programming codes used to generate them can be found in Supplemental Table 1.

### Statistical analysis

For the statistical analysis, appropriate methods were applied to analyze the data. Normally distributed continuous variables were expressed as mean ± SD, while non-normally distributed continuous variables were presented as medians and IQR. Categorical variables were reported as counts and percentages. To compare differences between groups, we used Pearson's chi-square test. The primary outcome of this study was cardiovascular mortality, with cancer mortality considered as a competing risk.

Participant characteristics were presented as counts and percentages, with differences between groups compared using Pearson’s chi-square test. Initially, univariate competing-risk analyses were performed, and cumulative-incidence function curves were plotted to visualize cardiovascular death changes for cancer survivors over time. To identify risk factors associated with cardiovascular mortality in cancer survivors, univariate and multivariate competing-risk models were employed. The multivariate models were adjusted for various variables: model 1 adjusted for sociodemographic factors, model 2 additionally included lifestyle characteristics, and model 3 further incorporated clinical characteristics. We subsequently applied the same strategy to examine CRP–cardiovascular-mortality associations within the major cancer subtypes. Cox proportional hazards regression was then used to estimate the influence of CRP on cardiovascular, all-cause, and cancer-specific mortality. Additionally, interactions on the multiplicative scale were tested using likelihood ratio tests, while interactions on the additive scale were evaluated by calculating the relative excess risk due to interaction.

Robustness was appraised in 4 sensitivity analyses: 1) exclusion of deaths occurring within the first 2 years of follow-up to minimize reverse causation; 2) categorization of CRP into clinically relevant strata (≤1, >1-<3, ≥3 mg/L); 3) division into population-based quartiles; and 4) modeling CRP as a continuous variable to characterize dose–response relationships with cardiovascular mortality.

A 2-sided *P* value of 0.05 was used to determine statistical significance. All statistical analysis was performed using R version 4.4.2 and RStudio Version 1.4.1717.

## Results

### Participant characteristics

The characteristics of all participants are provided in Table 1.

**Table 1:** Participant Characteristics

	CR P≤2 mg/L (n = 7,710)	CR P>2 mg/L (n = 7,710)	*P* Value
Sociodemographics			
Age, y	60.3 ± 6.8	60.3 ± 6.8	1.000
Sex			
Female	5,097 (66.1%)	5,097 (66.1%)	1.000
Male	2,613 (33.9%)	2,613 (33.9%)	
Ethnic			
White	7,538 (97.8%)	7,514 (97.5%)	0.225
Other races	172 (2.2%)	196 (2.5%)	
College or university degree			
No	5,150 (66.8%)	5,827 (75.6%)	<0.001
Yes	2,560 (33.2%)	1833 (24.4%)	
Lifestyle characteristics			
BMI, kg/m^2^	26.1 ± 4.0	29.6 ± 5.3	<0.001
Waist circumference, cm	86.4 ± 12.3	95.0 ± 13.4	<0.001
Number of times per week consuming processed meats			
None	836 (10.8%)	592 (7.7%)	<0.001
1-3	6,652 (86.3%)	6,852 (88.9%)	
4+	222 (2.9%)	266 (3.5%)	
Number of times per day consuming fruit or vegetables			
None	251 (3.3%)	373 (4.8%)	<0.001
1-2	3,283 (42.6%)	3,435 (44.6%)	
3-4	3,353 (43.5%)	3,146 (40.8%)	
5+	823 (10.7%)	756 (9.8%)	
Alcohol consumption			
Daily or almost daily	1,794 (23.3%)	1,407 (18.2%)	<0.001
3 or 4 times a week	1,779 (23.1%)	1,449 (18.8%)	
Once or twice a week	1,888 (24.5%)	1,942 (25.2%)	
1-3 times a month	774 (10.0%)	927 (12%)	
Special occasions only	876 (11.4%)	1,210 (15.7%)	
Never	599 (7.8%)	775 (10.1%)	
Smoke	4,726 (61.3%)	5,057 (65.6%)	<0.001
Days per week spent doing moderate physical activity >10 min			
None	889 (11.5%)	1,315 (17.1%)	<0.001
1-2	1,650 (21.4%)	1,649 (21.4%)	
3-7	5,171 (67.1%)	4,746 (61.6%)	
Clinical characteristics			
CRP, mg/L	0.9 (0.5-1.3)	3.8 (2.7-6.1)	<0.001
TC, mmol/L	5.7 ± 1.2	5.8 ± 1.2	<0.001
LDLC, mmol/L	3.5 ± 0.9	3.6 ± 0.9	<0.001
HDLC, mmol/L	1.5 ± 0.4	1.4 ± 0.4	<0.001
TG, mmol/L	1.6 ± 0.9	2.0 ± 1.1	<0.001
Family history of CVD	4,625 (60.0%)	4,521 (58.6%)	0.091
Hypertension	2,504 (32.5%)	3,177 (41.2%)	<0.001
Diabetes	439 (5.7%)	604 (7.8%)	<0.001
Hypercholesterolemia	1,455 (18.9%)	1,254 (16.3%)	<0.001
Coronary artery disease	470 (6.1%)	481 (6.2%)	0.738
Heart failure	6 (0.1%)	16 (0.2%)	0.055
Atrial fibrillation	85 (1.1%)	112 (1.5%)	0.062
Ischemic stroke	193 (2.5%)	218 (2.8%)	0.23
On antihypertensive medication	1,693 (22.0%)	1960 (25.4%)	<0.001
On statin	1,704 (22.1%)	1,560 (20.2%)	0.005
On diabetes medication	254 (3.3%)	331 (4.3%)	0.001
Chemotherapy	777 (10.1%)	1,065 (13.8%)	<0.001
Radiotherapy	176 (2.3%)	224 (2.9%)	0.017

BMI = body mass index; CRP = C-reactive protein; CVD = cardiovascular disease; HDL-C = high-density lipoprotein cholesterol; LDL-C = low-density lipoprotein cholesterol; TC = total cholesterol; TG = triglycerides.

Among all cancer survivors, 39.0% of participants had CRP >2 mg/L. Following propensity-score matching, the groups were well balanced with respect to age and sex. Participants with CRP >2 mg/L generally had lower educational levels and exhibited more unhealthy lifestyle characteristics, such as higher rates of obesity, processed meat consumption, limited fruit and vegetable intake, lack of exercise, and a higher smoking rate. Furthermore, the CRP >2 mg/L group had higher levels of TC, LDL-C, and TG, while their HDL-C levels were lower. This group also had a higher incidence of comorbidities, including diabetes, hypertension, and a history of chemotherapy and radiology. In contrast, the CRP ≤2 mg/L group had higher rates of alcohol consumption and hypercholesterolemia. As a result, more individuals in the CRP >2 mg/L group were on medications for hypertension and diabetes, while those in the CRP ≤2 mg/L group were more likely to be prescribed statins.

During an average follow-up of 10.2 ± 1.9 years, a total of 1,167 deaths occurred, equivalent to 7.6 deaths per 100 people. Of these, 103 (8.8%) were cardiovascular deaths and 813 (69.7%) were cancer-related deaths. The CRP >2 mg/L group accounted for 70 (68.0%) of cardiovascular deaths, while the CRP ≤2 mg/L group had 33 (32.0%). Compared to the CRP ≤2 mg/L group, the CRP >2 mg/L group showed a higher incidence of cardiovascular death.

### Associations of C-reactive protein with cardiovascular mortality in cancer survivors

**Figure 2:**
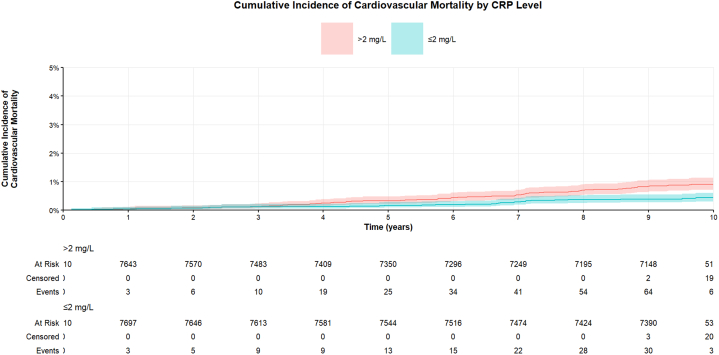
**Cumulative Incidence of Cardiovascular Mortality by C-Reactive Protein Level** Univariate competing-risk analyses were performed, and cumulative-incidence function curves were plotted to visualize cardiovascular death changes for cancer survivors over time. *P* <0.001. Abbreviation as in Figure 1.

Univariate analysis was conducted on cancer survivors, and cumulative incidence function curves were generated to compare differences between groups. The results revealed that CRP levels >2 mg/L were significantly associated with increased cardiovascular mortality in cancer survivors (sub-distribution hazard ratio [sHR]: 2.12; 95% CI: 1.4-3.21; *P* < 0.001) (Figure 2, Table 2).

**Table 2:** Subgroup Analysis

Stratified Variables	n	Percent	HR (95% CI)		*P* Value	*P* for Interaction
			CRP≤ 2 mg/L	CRP> 2 mg/L		
Overall	15,420	100	1.00	2.12 (1.40-3.21)	<0.001	
Age, y						0.399
<65	10,336	67.0%	1.00	1.78 (1.0-3.16)	0.051	
≥65	5,084	33.0%	1.00	2.54 (1.4-4.62)	0.002	
Sex						0.832
Female	10,194	66.10%	1.00	2.25 (1.14-4.44)	0.019	
Male	5,226	33.90%	1.00	2.05 (1.22-3.46)	0.007	
College or university degree						0.304
No	10,977	71.20%	1.00	1.84 (1.15-2.93)	0.011	
Yes	4,443	28.80%	1.00	3.11 (1.28-7.57)	0.012	
BMI, kg/m^2^						0.759
<30	11,171	72.40%	1.00	1.85 (1.12-3.05)	0.017	
≥30	4,249	27.60%	1.00	2.16 (0.91-5.14)	0.081	
Smoking history						0.043
No	5,637	36.6%	1.00	0.92 (0.38-2.22)	0.852	
Yes	9,783	63.4%	1.00	2.60 (1.59-4.23)	<0.001	
Days per week spent doing moderate physical activity >10 min						0.684
None	2,204	14.3	1.00	1.45 (0.59-3.54)	0.421	
1-2	3,299	21.4	1.00	2.44 (1.01-5.88)	0.047	
3-7	9,917	64.3	1.00	2.18 (1.26-3.78)	0.006	
LDL-C, mmol/L						0.207
Hypercholesterolemia	2,709	17.6%	1.00	1.26 (0.57-2.76)	0.565	
≥ 4.1	3,734	24.2%	1.00	1.63 (0.61-4.35)	0.328	
≥3.4-<4.1	4,014	26.0%	1.00	2.62 (1.03-6.69)	0.044	
≥2.6-<3.4	3,603	23.4%	1.00	7.29 (1.66-32.08)	0.009	
<2.6	1,360	8.8%	1.00	2.66 (1.09-6.53)	0.032	
HDL-C, mmol/L						0.606
<1	1,343	8.7%	1.00	1.58 (0.70-3.54)	0.269	
≥1	14,077	91.3%	1.00	2.02 (1.25-3.28)	0.004	
Hypertension						0.955
No	9,739	63.2%	1.00	1.94 (0.98-3.86)	0.057	
Yes	5,681	36.8%	1.00	1.90 (1.13-3.20)	0.016	
Diabetes						0.246
No	14,377	93.20%	1.00	2.29 (1.43-3.68)	0.001	
Yes	1,043	6.80%	1.00	1.28 (0.54-3.05)	0.581	
Coronary artery disease						0.150
No	14,469	93.80%	1.00	1.79 (1.13-2.84)	0.014	
Yes	951	6.20%	1.00	3.96 (1.49-10.56)	0.006	
Atrial fibrillation						0.319
No	15,223	98.70%	1.00	2.21 (1.44-3.40)	<0.001	
Yes	197	1.30%	1.00	1.00 (0.22-4.47)	1.00	
Ischemic stroke						0.949
No	15,009	97.30%	1.00	2.11 (1.37-3.23)	<0.001	
Yes	411	2.70%	1.00	2.23 (0.43-11.47)	0.339	
Chemotherapy						0.696
No	13,578	88.10%	1.00	2.02 (1.29-3.16)	0.002	
Yes	1842	11.90%	1.00	2.56 (0.84-7.76)	0.098	
Radiotherapy						0.344
No	15,020	97.40%	1.00	2.22 (1.44-3.41)	<0.001	
Yes	400	2.60%	1.00	1.04 (0.23-4.67)	0.955	

Abbreviations as in Table 1.

In model 1, after adjusting for sociodemographics, CRP >2 mg/L conferred a 1.08-fold excess risk of cardiovascular death in cancer survivors (sHR: 2.08; 95% CI: 1.37-3.16; *P* < 0.001) (Table 3). Furthermore, in model 2, which also adjusted for sociodemographic and lifestyle factors, CRP >2 mg/L remained significantly associated with a 72% higher risk of cardiovascular death (sHR: 1.72; 95% CI: 1.13-2.63; *P* = 0.012) (Table 4). After inclusion of clinical characteristics in model 3, CRP >2 mg/L continued to demonstrate a significant 64% increase in cardiovascular death risk (sHR: 1.64; 95% CI: 1.07-2.5; *P* = 0.023) (Table 5). These findings were corroborated in the fully adjusted Cox regression model, where CRP >2 mg/L remained independently associated with cardiovascular mortality (HR: 1.52; 95% CI: 1.31-1.77; *P* < 0.001) (Supplemental Table 2). Sensitivity analyses confirmed that the results were not materially altered by the Fine-Gray model’s truncation assumption.

**Table 3:** Associations Between CRP and Cardiovascular Mortality in Cancer Survivors for Model 1

	sHR	95% CI	*P* Value
Age, y			
<65	1.00		
≥65	1.85	1.24-2.75	0.002
Sex			
Female	1.00		
Male	2.99	1.99-4.50	<0.001
Ethnic			
White	1.00		
Other races	0.42	0.06-3.04	0.39
College or university degree			
No	1.00		
Yes	0.77	0.48-1.24	0.28
CRP, mg/L			
≤2	1.00		
>2	2.08	1.37-3.16	<0.001

sHR = sub-distribution hazard ratio; the other abbreviation as in Table 1.

**Table 4:** Associations Between CRP and Cardiovascular Mortality in Cancer Survivors for Model 2

	sHR	95% CI	*P* Value
Age, y			
<65	1.00		
≥65	1.95	1.30-2.92	0.001
Sex			
Female	1.00		
Male	2.80	1.79-4.39	<0.001
Ethnic			
White	1.00		
Other races	0.39	0.05-2.83	0.35
College or university degree			
No	1.00		
Yes	0.83	0.51-1.36	0.46
BMI, kg/m^2^			
<30	1.00		
≥30	1.39	0.88-2.17	0.15
Waist circumference, cm			
<90	1.00		
≥90	1.11	0.66-1.88	0.70
Number of times per week consuming processed meats			
None	1.00		
1-3	1.35	0.54-3.38	0.52
4+	0.71	0.17-3.04	0.65
Number of times per day consuming fruit or vegetables			
None	1.00		
1-2	0.70	0.33-1.49	0.36
3-4	0.55	0.25-1.18	0.12
5+	0.47	0.17-1.29	0.14
Alcohol consumption			
Daily or almost daily	1.00		
3 or 4 times a week	0.60	0.32-1.13	0.11
Once or twice a week	0.72	0.40-1.28	0.26
1-3 times a month	1.33	0.70-2.51	0.38
Special occasions only	0.76	0.36-1.62	0.49
Never	1.85	0.96-3.55	0.065
Smoking history			
No	1.00		
Yes	2.07	1.26-3.42	0.004
Days per week spent doing moderate physical activity >10 min			
None	1.00		
1-2	0.92	0.50-1.72	0.80
3-7	0.71	0.42-1.20	0.20
CRP, mg/L			
≤2	1.00		
>2	1.72	1.13-2.63	0.012

Abbreviations as in Table 1.

**Table 5:** Associations Between CRP and Cardiovascular Mortality in Cancer Survivors for Model 3

	HR	95% CI	*P* Value
Age, y			
<65	1.00		
≥65	1.70	1.12-2.57	0.013
Sex			
Female	1.00		
Male	1.94	1.16-3.26	0.012
Ethnic			
White	1.00		
Other races	0.38	0.05-2.77	0.34
College or university degree			
No	1.00		
Yes	0.90	0.55-1.46	0.66
BMI, kg/m^2^			
<30	1.00		
≥30	1.06	0.66-1.71	0.80
Waist circumference, cm			
<90	1.00		
≥90	0.91	0.52-1.58	0.73
Number of times per week consuming processed meats			
None	1.00		
1-3	1.29	0.52-3.23	0.58
4+	0.65	0.15-2.80	0.56
Number of times per day consuming fruit or vegetables			
None	1.00		
1-2	0.71	0.32-1.56	0.39
3-4	0.55	0.25-1.22	0.14
5+	0.45	0.16-1.26	0.13
Alcohol consumption			
Daily or almost daily	1.00		
3 or 4 times a week	0.58	0.31-1.09	0.089
Once or twice a week	0.63	0.35-1.14	0.13
1-3 times a month	1.07	0.58-1.99	0.83
Special occasions only	0.58	0.27-1.26	0.17
Never	1.24	0.63-2.42	0.54
Smoking history			
No	1.00		
Yes	1.91	1.17-3.14	0.01
Days per week spent doing moderate physical activity >10 min			
None	1.00		
1-2	1.04	0.54-1.99	0.91
3-7	0.85	0.49-1.48	0.57
LDL-C, mmol/L			
Hypercholesterolemia	1.00		
≥ 4.1	1.59	0.70-3.63	0.27
≥3.4-<4.1	1.53	0.71-3.28	0.27
≥2.6-<3.4	0.94	0.45-1.98	0.88
<2.6	1.76	0.93-3.32	0.08
HDL-C, mmol/L			
<1	1.00		
≥1	0.53	0.32-0.86	0.011
TG, mmol/L			
≥2.3	1.00		
≥1.7-<2.3	0.92	0.53-1.58	0.75
<1.7	0.96	0.60-1.55	0.88
Family history of cardiovascular disease			
No	1.00		
Yes	0.88	0.59-1.33	0.55
Hypertension			
No	1.00		
Yes	2.17	1.24-3.79	0.007
Diabetes			
No	1.00		
Yes	1.49	0.71-3.11	0.29
Coronary artery disease			
No	1.00		
Yes	2.08	1.19-3.64	0.01
Heart failure			
No	1.00		
Yes	5.81	0.77-44.0	0.089
Atrial fibrillation			
No	1.00		
Yes	3.11	1.35-7.15	0.008
Ischemic stroke			
No	1.00		
Yes	1.29	0.55-3.01	0.56
On antihypertensive medication			
Nonuser	1.00		
User	1.06	0.62-1.83	0.83
On statin			
Nonuser	1.00		
User	1.38	0.72-2.63	0.33
On diabetes medication			
Nonuser	1.00		
User	1.04	0.44-2.45	0.93
Chemotherapy			
No	1.00		
Yes	1.59	0.94-2.68	0.081
Radiotherapy			
No	1.00		
Yes	2.11	0.93-4.80	0.075
CRP, mg/L			
≤2	1.00		
>2	1.64	1.07-2.50	0.023

Abbreviations as in Table 1.

The relationship between CRP and all-cause mortality and cancer-related mortality in cancer survivors is shown in Supplemental Tables 3, 4, Supplemental Figures 1 and 2.

Among 8 common cancer subtypes, we examined the association between CRP and cardiovascular mortality. Lung, prostate, and bladder cancers were excluded because certain covariates exhibited no within-covariate variability, precluding reliable estimation. In model 1 (sociodemographics), CRP >2 mg/L was associated with higher cardiovascular mortality among survivors of breast, skin, and lymphoid malignancies. The association endured through model 2 (lifestyle factors) for these 3 malignancies. In the fully adjusted model (model 3), point estimates remained elevated for breast and skin cancers, yet neither attained statistical significance. Conversely, colorectal cancer displayed a consistent inverse association across all models, and cervical cancer also showed a protective trend, achieving conventional significance in model 2 (Supplemental Table 5).

### Subgroup analyses

As shown in Table 4, the association between CRP >2 mg/L and an increased risk of cardiovascular death was observed across cancer survivors. Notably, this relationship appeared to be more pronounced among smokers. These subgroups encompassed various age ranges, sex, educational backgrounds (college or university education), BMI, smoking history, LDL-C and HDL-C levels, as well as the presence of comorbidities such as hypertension, diabetes, CAD, atrial fibrillation, ischemic stroke, and a history of chemotherapy and radiotherapy (Table 2). This suggests that CRP may serve as an independent predictor of cardiovascular mortality in a broad spectrum of cancer survivors, particularly among smokers.

### Sensitivity analyses

To mitigate reverse causation, we first excluded 243 survivors who died within 2 years of enrollment. Competing-risk analysis of the remaining cohort confirmed that baseline CRP >2 mg/L conferred an 83% higher subdistribution hazard of cardiovascular death (sHR: 1.83; 95% CI: 1.15-2.89; *P* = 0.01) (Supplemental Table 6).

When CRP was stratified by clinically relevant cut points, both intermediate (1-3 mg/L) and high (≥3 mg/L) levels were associated with excess risk relative to ≤1 mg/L:

• 1 to 3 mg/L: sHR: 1.56 (95% CI: 0.85-2.87; *P* = 0.15)
• ≥3 mg/L: sHR: 1.99 (95% CI: 1.90-3.66; *P* = 0.026) (Supplemental Table 7).

**Figure 3:**
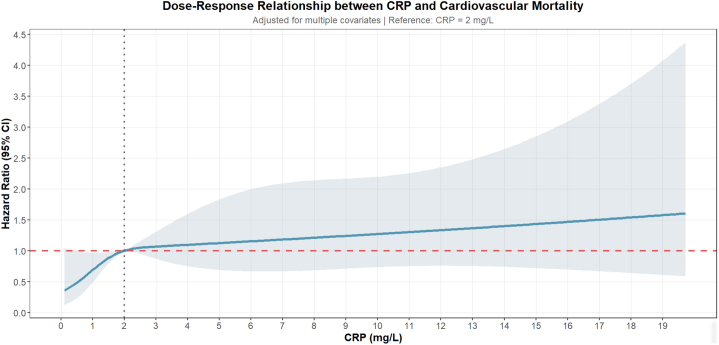
**Dose–Response Relationship Between C-Reactive Protein Level and Cardiovascular Mortality** Restricted cubic-spline (4 knots at 5th, 35th, 65th, 95th percentiles) illustrating the multivariable-adjusted, nonlinear dose–response relationship between continuous CRP levels and HR for CV mortality; shaded area represents 95% confidence band. Reference (HR = 1.0) set at CRP = 2 mg/L. Abbreviation as in Figure 1.

Quartile analysis revealed a monotonic increase in cardiovascular mortality across CRP distributions: survivors in Q3-Q4 experienced significantly higher risk than those in the lowest quartile (Q1) (Supplemental Table 8). Restricted cubic-spline modeling indicated a threshold effect, with rising CRP concentrations translating into greater cardiovascular hazard from 2 mg/L upward (Figure 3).

## Discussion

To our knowledge, this is the first and largest population-based cohort study to examine the link between chronic inflammation and cardiovascular mortality in cancer survivors across all cancer types. Our findings demonstrate a significant and independent association between chronic inflammation and increased risk of cardiovascular death. Notably, this association was more pronounced among smokers. Importantly, the relationship remained consistent regardless of age, sex, BMI, smoking, physical activity, and the presence of hypertension, diabetes, CAD, atrial fibrillation, ischemic stroke, and a history of chemotherapy and radiotherapy (Central Illustration).

**Central Illustration:**
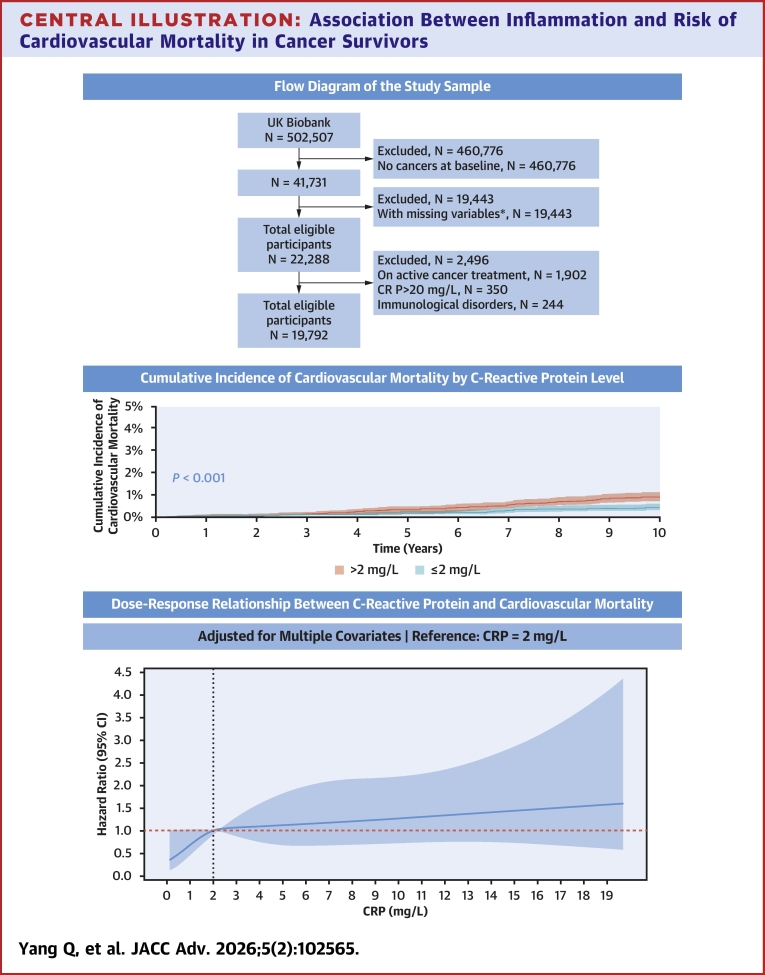
**Association Between Inflammation and Risk of Cardiovascular Mortality in Cancer Survivors** Top panel: Flow diagram of the study population, including application of inclusion/exclusion criteria and final sample size available for analysis. Middle panel: Cumulative incidence of cardiovascular mortality by C-reactive protein level, estimated with Gray’s test for competing events (cancer death) *P* <0.001. Bottom panel: Restricted cubic spline (4 knots at 5th, 35th, 65th, 95th percentiles) illustrating the multivariable-adjusted, nonlinear dose–response relationship between continuous C-reactive protein levels and HR for cardiovascular mortality; shaded area represents 95% confidence band. Reference (HR: 1.00) set at CRP = 2 mg/L. Abbreviation as in Figure 1.

Our study also highlights the high prevalence of common comorbidities in cancer survivors. Individuals with CRP levels >2 mg/L were more likely to have hypertension, diabetes, heart failure, atrial fibrillation, and ischemic stroke—conditions known to increase cardiovascular mortality risk.^2^ Our analysis confirmed that hypertension, CAD, and atrial fibrillation were independent risk factors for cardiovascular mortality. However, even after adjusting for preexisting cardiovascular conditions, elevated CRP levels remained a significant predictor of cardiovascular mortality, underscoring the important role of inflammation in this population.

Consistent with the findings of Saro H. et al,^18^ our study confirmed that cancer remained the leading cause of death among cancer survivors after 10 years, accounting for 69.7% of all deaths. However, CVD also emerged as a significant contributor, responsible for 8.8% of deaths in this population.

Our findings suggest that CRP levels >2 mg/L are associated with an increased risk of cardiovascular death in cancer survivors, aligning with some—but not all—previous studies. CRP, a marker of systemic inflammation, is prevalent among cancer patients.^19^ In our study, 39.0% of cancer survivors had CRP >2.0 mg/L, a common threshold for low-grade inflammation.^11^^,^^15^^,^^16^ Prior research has linked inflammation to cardiovascular mortality in the general population,^8^ in healthy women,^9^ and in patients with CVD.^10^^,^^11^ In a recent cohort of 84,399 adults with established atherosclerotic cardiovascular disease, Faizan Mazhar et al observed that nearly three-fifths had CRP levels >2 mg/L; these individuals experienced a 35% higher all-cause mortality risk than those with CRP ≤2 mg/L, underscoring the prognostic weight of this inflammatory threshold.^11^ The prevalence of CRP >2 mg/L in our cancer-survivor cohort was 39%—substantially lower than the ≈60% reported by Mazhar—likely reflecting differences in baseline characteristics, prior cancer therapies, or immune-modulating effects of malignancy itself. Clinical trials have also shown that anti-inflammatory therapies, such as canakinumab and low-dose colchicine, can reduce major adverse cardiovascular events, including cardiovascular death, in patients with CAD.^15^^,^^20^ However, in patients with heart failure, various anti-inflammatory treatments—including tumor necrosis factor-α inhibitors, interleukin (IL)-1 receptor antagonists, methotrexate, and colchicine—have not demonstrated mortality benefits.^21^

Inflammation is a common risk factor for both cancer and CVD,^22^ with evidence suggesting it contributes to the development of both conditions.^23^ The CANTOS (The Canakinumab Anti-Inflammatory Thrombosis Outcomes Study) trial demonstrated that IL-1β inhibition not only reduced cardiovascular mortality but also lowered lung cancer incidence.^12^^,^^15^ Additionally, elevated CRP has been associated with increased all-cause and cancer-related mortality in cancer patients.^4^, ^5^, ^6^, ^7^ Despite these insights, there is a gap in research regarding the relationship between CRP and cardiovascular mortality in cancer survivors. A study by Brandon et al found that CRP was linked to long-term all-cause mortality in breast cancer patients, though this was not attributed to CVD—possibly because their study excluded individuals with a history of cardiovascular events.^7^ Our results suggest that chronic inflammation, as reflected by CRP, is an important and independent risk factor for cardiovascular mortality in cancer survivors. This highlights CRP’s potential as a biomarker for monitoring cancer therapy-related cardiovascular toxicity and underscores the need for targeted anti-inflammatory interventions to mitigate cardiovascular risk.

### Strengths and limitations

Our study has several notable strengths that enhance the reliability of its findings. Firstly, it utilized the UK Biobank database, which is large, comprehensive, and well-regarded, with an extensive follow-up period. This robust data set helps mitigate common biases often encountered in observational studies, such as selection and recall bias. To account for the potential impact of CVD risk factors and a history of CVD on cardiovascular mortality, we included these variables in our analysis. Importantly, our results demonstrate that the association between CRP levels and increased cardiovascular mortality risk in cancer survivors remained independent of baseline comorbid CVD. This suggests that our conclusions may be applicable to a broader range of cancer survivors.

However, there are also limitations to our study. Despite including variables that account for common cardiovascular risk factors in cancer survivors, there may still be unmeasured confounders that were not considered. Furthermore, the sample sizes within each cancer subtype were small, some variables were present at only one level, and detailed radiotherapy and chemotherapy regimens were lacking. Additionally, while efforts were made to ensure a diverse sample, the cohort was predominantly composed of Caucasians, and the potential influence of race should be considered when interpreting the study’s findings. Finally, the multiple comparisons performed in sensitivity analyses raise the probability of type I error, and the wide CIs seen in a limited number of associations indicate that some analyses may be underpowered.

## Conclusions

This study demonstrates that chronic inflammation, indicated by elevated CRP levels, is significantly associated with the risk of cardiovascular mortality in survivors of various cancer types. Future research should focus on whether targeting inflammatory pathways can reduce the burden of cardiovascular mortality in this population.

Perspectives **COMPETENCY IN MEDICAL KNOWLEDGE:** This proof-of-concept study provides compelling evidence that systemic inflammation constitutes an independent and modifiable risk factor for cardiovascular mortality among cancer survivors.

**TRANSLATIONAL OUTLOOK:** These findings catalyze a paradigm shift toward inflammation-targeted interventions as a strategy to mitigate late cardiovascular sequelae in the rapidly growing population of cancer survivors. Prospective clinical trials are now warranted to determine whether selective anti-inflammatory agents—alone or integrated with cardio-oncology care pathways—can durably attenuate this risk, thereby transforming tertiary prevention and closing the translational gap between tumor control and cardiovascular health.

## Funding support and author disclosures

This work was supported by grants from 10.13039/501100012151Sanming Project of Medicine in Shenzhen (No.SZSM202411021 and No.SZSM202311022) and Shenzhen Clinical Research Center for Rare Diseases (LCYSSQ20220823091402005). The authors have reported that they have no relationships relevant to the contents of this paper to disclose.
