# Supplementary material for: Association Between Inflammation and Risk of Cardiovascular Mortality in Cancer Survivors
Source: JACC Adv. 2026 Jan 23;5(2):102565. doi: 10.1016/j.jacadv.2025.102565 (PMC12860934; doi:10.1016/j.jacadv.2025.102565)
Supplement: Supplemental Tables 1-8 and Supplemental Figures 1, 2 [file mmc1.pdf]

Table S1.Codelists for Outcomes, Exposure, and Covariates

Codelists for outcomes, exposure, and covariates	UKB Data-Field Number	Description or ICD10 code
Age	n_21003 _*	This is a derived variable based on date of birth and date of attending assessment centre and refers to the age of the participant on the day they attended an Assessment Centre, truncated to whole year part.
Date of attending assessment centre	s_53_*	Date when a participant attended a UK Biobank assessment centre.
Sex	n_31_*	Sex of participant.
Ethnic	n_21000_*	This is an amalgam of sequential branching questions asked during the initial Assessment Centre visit as part of the touchscreen questionnaire.
College or university degree	n_6138_*	ACE touchscreen question "Which of the following qualifications do you have?
BMI	n_21001_*	Constructed from height and weight measured during the initial Assessment Centre visit
Waist circumference	n_48_*	Waist circumference
Number of times per week consuming processed meats	n_1349_*	ACE touchscreen question "How often do you eat processed meats (such as bacon, ham, sausages, meat pies, kebabs, burgers, chicken nuggets)?"
Number of times per day consuming fruit or vegetables	n_1289_*	ACE touchscreen question "On average how many heaped tablespoons of COOKED vegetables would you eat per DAY? (Do not include potatoes; put '0' if you do not eat any)"
Alcohol consumption	n_1558_*	ACE touchscreen question "About how often do you drink alcohol?"
Ever smoked	n_20160_*	Derived using variables "Current tobacco smoking" (Field 1239) and "Past tobacco smoking" (Field 1249).
C-reactive protein	n_30710_*	Measured by immunoturbidimetric - high sensitivity analysis on a Beckman Coulter AU5800
LDL-C	n_30780_*	Measured by enzymatic protective selection analysis on a Beckman Coulter AU5800
HDL-C	n_30760_*	Measured by enzyme immunoinhibition analysis on a Beckman Coulter AU5800
TG	n_30870_*	Measured by GPO-POD analysis on a Beckman Coulter AU5800
Family history of cardiovascular disease	n_20110_*<br>n_20107_*	Illnesses of mother family history<br>Illnesses of father family history

	n_20111_*	Illnesses of siblings family history
Diabetes	n_20002_*	1220、1222、1223
Hypertension	n_20002_*,	1065、1072
Hypercholesterolaemia	s_41270_*	E780、E782、E783、E784、E785
Coronary artery disease	n_20002_*	1074、1075
Heart failure	n_20002_*	1076
Atrial fibrillation	n_20002_*	1471
Ischaemic stroke	n_20002_*	1081、1082
Immunological disorders	n_20002_*	1371、1372、1373、1376、1377、1378、1379、1380、1381、1382、1383、1384、1561
On antihypertensive medication	n_20003_*	Code for treatment. Negative codes indicate free-text entry.
On statin	n_20003_*	Code for treatment. Negative codes indicate free-text entry.
On diabetes medication	n_20003_*	Code for treatment. Negative codes indicate free-text entry.
Chemotherapy	s_41270_0_	Z511,Z512
Radiotherapy	s_41270_0_	Z510
Self-reported Cancer	n_20001_*	Code for cancer. If the participant was uncertain of the type of cancer they had had, then they described it to the interviewer (a trained nurse) who attempted to place it within the coding tree. If the cancer could not be located in the coding tree then the interviewer entered a free-text description of it. These free-text descriptions were subsequently examined by a doctor and, where possible, matched to entries in the coding tree. Free-text descriptions which could not be matched with very high probability have been marked as "unclassifiable".
All cause death	n_40018_*	Indicates the format of the data in which the original death record was supplied to UK Biobank.
Cardiovascular death	s_40001_*	Indicates the format of the data in which the original death record was supplied to UK Biobank.

Cancer death	s_40001_*	Indicates the format of the data in which the original death record was supplied to UK Biobank.
Date of all cause death	s_40000_*	Acquired from central registry.
Date of cardiovascular death	s_40000_*	Acquired from central registry.
Date of cancer death	s_40000_*	Acquired from central registry.
For further details, please visit the UK Biobank Data Showcase at https://biobank.ndph.ox.ac.uk/showcase/index.cgi. The UKB Data-Field numbers above can be searched in the Data Showcase to access highly detailed notes about instruments used,validation exercises (where applicable), screenshots of the survey, coding of the variable, and summary statistics of each variable.		

Table S2.Cox Regression Analysis Results for Cardiovascular Mortality in Model 3

	Univariable			Multivariable		
	HR	95%CI	P value	HR	95%CI	P value
Age, years						
<65	1			1		
≥65	1.32	1.15-1.52	p<0.001	1.41	1.22-1.64	p<0.001
Sex						
Female	1			1		
Male	1.64	1.43-1.88	p<0.001	1.68	1.42-1.98	p<0.001
Ethnic						
White	1					
Other races	1.21	0.80-1.83	p=0.368			
College or university degree						
No	1			1		
Yes	0.81	0.69-0.95	p=0.011	0.91	0.77-1.07	p=0.252
BMI, kg/m^2						
<30	1			1		
≥30	1.16	1.00-1.35	p=0.045	0.97	0.81-1.17	p=0.779
Waist circumference, cm						
<90	1			1		
≥90	1.39	1.20-1.59	p<0.001	0.91	0.75-1.09	p=0.308
Number of times per week consuming processed meats						
None	1					
1~3	0.99	0.78-1.25	p=0.906			
4+	1.17	0.76-1.78	p=0.478			
Number of times per day consuming fruit or vegetables						
None	1			1		
1~2	0.81	0.59-1.12	p=0.196	1.08	0.78-1.50	p=0.651
3~4	0.78	0.57-1.08	p=0.138	1.05	0.75-1.46	p=0.773
5+	0.73	0.50-1.06	p=0.099	0.97	0.66-1.43	p=0.892
Alcohol consumption						
Daily or almost daily	1			1		
Three or four times a week	0.84	0.67-1.05	p=0.127	0.87	0.69-1.08	p=0.204
Once or twice a week	0.93	0.75-1.14	p=0.483	0.86	0.70-1.07	p=0.169
One to three times a month	0.89	0.69-1.17	p=0.410	0.82	0.63-1.08	p=0.162
Special occasions only	1.25	1.00-1.57	p=0.049	1.21	0.96-1.53	p=0.112
Never	1.24	0.96-1.61	p=0.094	1.07	0.82-1.40	p=0.612
Smoking history						
No	1			1		
Yes	1.10	0.96-1.28	p=0.178	1.03	0.89-1.20	p=0.677
Days per week spent doing moderate physical activity >10 mins						
None	1			1		
1~2	0.52	0.42-0.65	p<0.001	0.61	0.49-0.76	p<0.001

3~7	0.59	0.50-0.70	p<0.001	0.70	0.58-0.83	p<0.001
LDLC, mmol/L						
Hypercholesterolaemia	1			1		
≥ 4.1	1.10	0.89-1.37	p=0.358	1.22	0.92-1.62	p=0.165
≥3.4<4.1	0.91	0.74-1.14	p=0.418	0.99	0.75-1.31	p=0.940
≥2.6<3.4	0.96	0.77-1.20	p=0.732	1.01	0.77-1.31	p=0.963
<2.6	1.24	0.95-1.62	p=0.114	1.09	0.82-1.45	p=0.549
HDLC, mmol/L						
<1	1			1		
≥1	0.59	0.48-0.72	p<0.001	0.81	0.64-1.01	p=0.063
TG, mmol/L						
≥2.3	1			1		
≥1.7<2.3	0.86	0.71-1.05	p=0.148	0.97	0.80-1.19	p=0.776
<1.7	0.76	0.65-0.90	p=0.001	1.03	0.86-1.23	p=0.763
Family history of cardiovascular disease						
No	1					
Yes	0.95	0.83-1.10	p=0.516			
Hypertension						
No	1					
Yes	1.05	0.91-1.20	p=0.539			
Diabetes						
No	1			1		
Yes	1.46	1.15-1.84	p=0.002	0.92	0.62-1.36	p=0.662
Coronary artery disease						
No	1			1		
Yes	1.52	1.20-1.93	p<0.001	1.23	0.95-1.61	p=0.118
Heart failure						
No	1					
Yes	1.75	0.44-7.02	p=0.428			
Atrial fibrillation						
No	1					
Yes	0.67	0.32-1.40	p=0.287			
Ischaemic stroke						
No	1			1		
Yes	2.07	1.52-2.82	p<0.001	1.82	1.33-2.50	p<0.001
On antihypertensive medication						
Nonuser	1			1		
User	1.12	0.95-1.31	p=0.174	0.91	0.76-1.09	p=0.296
On statin						
Nonuser	1			1		
User	1.15	0.98-1.36	p=0.083	0.97	0.76-1.24	p=0.809
On diabetes medication						
Nonuser	1			1		
User	1.69	1.27-2.25	p<0.001	1.44	0.90-2.30	p=0.126

Chemotherapy						
No	1			1		
Yes	5.40	4.69-6.22	p<0.001	5.31	4.59-6.13	p<0.001
Radiotherapy						
No	1			1		
Yes	2.63	1.98-3.50	p<0.001	1.49	1.11-1.99	p=0.007
CRP, mg/L						
≤2	1			1		
>2	1.73	1.50-2.00	p<0.001	1.52	1.31-1.77	p<0.001

Table S3. Cox Regression Analysis Results for All Cause Mortality in Model 3

	Univariable			Multivariable		
	HR	95%CI	P value	HR	95%CI	P value
Age, years						
<65	1			1		
≥65	1.52	1.35-1.71	p<0.001	1.59	1.34-1.71	p<0.001
Sex						
Female	1			1		
Male	1.93	1.72-2.17	p<0.001	1.80	1.56-2.07	p<0.001
Ethnic						
White	1					
Other races	1.10	0.76-1.58	p=0.620			
College or university degree						
No	1			1		
Yes	0.75	0.65-0.86	p<0.001	0.85	0.74-0.98	p=0.027
BMI, kg/m^2						
<30	1			1		
≥30	1.29	1.14-1.46	p<0.001	1.04	0.89-1.20	p=0.635
Waist circumference, cm						
<90	1			1		
≥90	1.57	1.39-1.77	p<0.001	0.92	0.78-1.07	p=0.276
Number of times per week consuming processed meats						
None	1			1		
1~3	1.08	0.88-1.32	p=0.485	0.96	0.78-1.19	p=0.725
4+	1.63	1.17-2.27	p=0.004	1.14	0.81-1.59	p=0.461
Number of times per day consuming fruit or vegetables						
None	1			1		
1~2	0.65	0.51-0.83	p<0.001	0.89	0.69-1.15	p=0.372
3~4	0.61	0.48-0.78	p<0.001	0.83	0.65-1.08	p=0.166
5+	0.61	0.45-0.82	p<0.001	0.81	0.60-1.10	p=0.184
Alcohol consumption						
Daily or almost daily	1			1		
Three or four times a week	0.74	0.62-0.89	p=0.002	0.78	0.64-0.93	p=0.008
Once or twice a week	0.85	0.72-1.01	p=0.065	0.83	0.70-0.99	p=0.039
One to three times a month	0.82	0.66-1.03	p=0.085	0.80	0.64-1.00	p=0.052
Special occasions only	1.07	0.89-1.3	p=0.464	1.07	0.87-1.30	p=0.527
Never	1.31	1.07-1.60	p=0.01	1.13	0.91-1.41	p=0.250
Smoking history						
No	1			1		
Yes	1.26	1.11-1.43	p<0.001	1.13	1.00-1.29	p=0.056
Days per week spent doing moderate physical activity >10mins						
None	1			1		
1~2	0.54	0.45-0.64	p<0.001	0.66	0.55-0.79	p<0.001
3~7	0.57	0.49-0.65	p<0.001	0.69	0.60-0.80	p<0.001

LDLC, mmol/L						
Hypercholesterolaemia	1			1		
≥ 4.1	0.94	0.79-1.13	p=0.516	1.31	1.03-1.66	p=0.026
≥3.4<4.1	0.88	0.74-1.05	p=0.168	1.17	0.93-1.48	p=0.177
≥2.6<3.4	0.98	0.82-1.17	p=0.792	1.19	0.96-1.48	p=0.105
<2.6	1.32	1.07-1.64	p=0.011	1.23	0.98-1.55	p=0.069
HDLC, mmol/L						
<1	1			1		
≥1	0.52	0.44-0.61	p<0.001	0.79	0.66-0.95	p=0.013
TG, mmol/L						
≥2.3	1			1		
≥1.7<2.3	0.89	0.75-1.04	p=0.146	1.02	0.87-1.21	p=0.795
<1.7	0.77	0.67-0.88	p<0.001	1.09	0.94-1.27	p=0.252
Family history of cardiovascular disease						
No	1					
Yes	0.96	0.86-1.08	p=0.543			
Hypertension						
No	1			1		
Yes	1.29	1.15-1.45	p<0.001	1.09	0.94-1.26	p=0.258
Diabetes						
No	1			1		
Yes	1.79	1.50-2.15	p<0.001	1.11	0.83-1.48	p=0.481
Coronary artery disease						
No	1			1		
Yes	2.11	1.76-2.52	p<0.001	1.49	1.22-1.82	p<0.001
Heart failure						
No	1			1		
Yes	2.50	0.94-6.67	p=0.068	2.49	0.93-6.67	p=0.070
Atrial fibrillation						
No	1			1		
Yes	1.44	0.93-2.21	p=0.10	1.06	0.69-1.64	p=0.783
Ischaemic stroke						
No	1			1		
Yes	2.31	1.80-2.95	p<0.001	1.81	1.41-2.34	p<0.001
On antihypertensive medication						
Nonuser	1			1		
User	1.44	1.28-1.61	p<0.001	0.97	0.83-1.13	p=0.683
On statin						
Nonuser	1			1		
User	1.38	1.22-1.57	p<0.001	0.95	0.80-1.12	p=0.571
On diabetes medication						
Nonuser	1			1		
User	1.91	1.52-2.40	p<0.001	1.17	0.83-1.67	p=0.372
Chemotherapy						

No	1			1		
Yes	3.87	3.42-4.38	p<0.001	3.84	3.38-4.35	p<0.001
Radiotherapy						
No	1			1		
Yes	2.35	1.83-3.02	p<0.001	1.51	1.17-1.96	p=0.001
CRP, mg/L						
≤2	1			1		
>2	1.84	1.63-2.07	p<0.001	1.60	1.41-1.82	p<0.001

Table S4. Cox Regression Analysis Results for Cancer Mortality in Model 3

	Univariable			Multivariable		
	HR	95%CI	P value	HR	95%CI	P value
Age, years						
<65	1			1		
≥65	1.32	1.15-1.52	p<0.001	1.41	1.22-1.64	p<0.001
Sex						
Female	1			1		
Male	1.64	1.43-1.88	p<0.001	1.68	1.42-1.98	p<0.001
Ethnic						
White	1					
Other races	1.21	0.80-1.83	p=0.368			
College or university degree						
No	1			1		
Yes	0.81	0.69-0.95	p=0.011	0.91	0.77-1.07	p=0.252
BMI, kg/m^2						
<30	1			1		
≥30	1.16	1.00-1.35	p=0.045	0.97	0.81-1.17	p=0.779
Waist circumference, cm						
<90	1			1		
≥90	1.39	1.20-1.59	p<0.001	0.91	0.75-1.09	p=0.308
Number of times per week consuming processed meats						
None	1					
1~3	0.99	0.78-1.25	p=0.906			
4+	1.17	0.76-1.78	p=0.478			
Number of times per day consuming fruit or vegetables						
None	1			1		
1~2	0.81	0.59-1.12	p=0.196	1.08	0.78-1.50	p=0.651
3~4	0.78	0.57-1.08	p=0.138	1.05	0.75-1.46	p=0.773
5+	0.73	0.50-1.06	p=0.099	0.97	0.66-1.43	p=0.892
Alcohol consumption						
Daily or almost daily	1			1		
Three or four times a week	0.84	0.67-1.05	p=0.127	0.87	0.69-1.08	p=0.204
Once or twice a week	0.93	0.75-1.14	p=0.483	0.86	0.70-1.07	p=0.169
One to three times a month	0.89	0.69-1.17	p=0.410	0.82	0.63-1.08	p=0.162
Special occasions only	1.25	1.00-1.57	p=0.049	1.21	0.96-1.53	p=0.112
Never	1.24	0.96-1.61	p=0.094	1.07	0.82-1.40	p=0.612
Smoking history						
No	1					
Yes	1.10	0.96-1.28	p=0.178	1.03	0.89-1.20	p=0.677
Days per week spent doing moderate physical activity >10 mins						
None	1			1		
1~2	0.52	0.42-0.65	p<0.001	0.61	0.49-0.76	p<0.001
3~7	0.59	0.50-0.70	p<0.001	0.70	0.58-0.83	p<0.001

LDLC						
Hypercholesterolaemia	1			1		
≥ 4.1	1.10	0.89-1.37	p=0.358	1.22	0.92-1.62	p=0.165
≥3.4<4.1	0.91	0.74-1.14	p=0.418	0.99	0.75-1.31	p=0.940
≥2.6<3.4	0.96	0.77-1.20	p=0.732	1.01	0.77-1.31	p=0.963
<2.6	1.24	0.95-1.62	p=0.114	1.09	0.82-1.45	p=0.549
HDLC						
<1	1			1		
≥1	0.59	0.48-0.72	p<0.001	0.81	0.64-1.01	p=0.063
TG						
≥2.3	1			1		
≥1.7<2.3	0.86	0.71-1.05	p=0.148	0.97	0.80-1.19	p=0.776
<1.7	0.76	0.65-0.90	p=0.001	1.03	0.86-1.23	p=0.763
Family history of cardiovascular disease						
No	1					
Yes	0.95	0.83-1.10	p=0.516			
Hypertension						
No	1					
Yes	1.05	0.91-1.20	p=0.539			
Diabetes						
No	1			1		
Yes	1.46	1.15-1.84	p=0.002	0.92	0.62-1.36	p=0.662
Coronary artery disease						
No	1			1		
Yes	1.52	1.20-1.93	p<0.001	1.23	0.95-1.61	p=0.118
Heart failure						
No	1					
Yes	1.75	0.44-7.02	p=0.428			
Atrial fibrillation						
No						
Yes	0.67	0.32-1.40	p=0.287			
Ischaemic stroke						
No	1			1		
Yes	2.07	1.52-2.82	p<0.001	1.82	1.33-2.50	p<0.001
On antihypertensive medication						
Nonuser	1			1		
User	1.12	0.95-1.31	p=0.174	0.91	0.76-1.09	p=0.296
On statin						
Nonuser	1			1		
User	1.15	0.98-1.36	p=0.083	0.97	0.76-1.24	p=0.809
On diabetes medication						
Nonuser	1			1		
User	1.69	1.27-2.25	p<0.001	1.44	0.90-2.30	p=0.126
Chemotherapy						

No	1			1		
Yes	5.40	4.69-6.22	p<0.001	5.31	4.59-6.13	p<0.001
Radiotherapy						
No	1			1		
Yes	2.63	1.98-3.50	p<0.001	1.49	1.11-1.99	p=0.007
CRP, mg/L						
≤2	1			1		
>2	1.73	1.50-2.00	p<0.001	1.52	1.31-1.77	p<0.001

Table S5. Associations Between CRP and Cardiovascular Mortality in Cancer Subtypes

Cancer subtypes	Model1			Model2			Model3		
	sHR	95%CI	P-value	sHR	95%CI	P-value	sHR	95%CI	P-value
Lung cancer	NA	NA	NA	NA	NA	NA	NA	NA	NA
Breast cancer	3.68	0.99-13.7	0.052	3.08	0.79-11.9	0.10	3.38	0.66-17.3	0.14
Skin cancer	1.35	0.54-3.35	0.52	1.36	0.56-3.29	0.50	1.23	0.34-4.44	0.76
Colorectal cancer	0.91	0.28-2.97	0.87	0.79	0.24-2.61	0.70	0.83	0.18-3.80	0.81
Prostate cancer	NA	NA	NA	NA	NA	NA	NA	NA	NA
Cervical cancer	0.33	0.03-3.69	0.37	0.08	0.01-0.61	0.014	NA	NA	NA
Bladder cancer	NA	NA	NA	NA	NA	NA	NA	NA	NA
Lymphoma	1.85	0.35-9.84	0.47	1.83	0.47-7.17	0.39	NA	NA	NA

Table S6. Associations Between CRP and Cardiovascular Mortality in Cancer Survivors for Model 3 Excluding Deaths within the First 2 years

	sHR	95%CI	P value
Age, years			
<65	1		
≥65	1.68	1.08-2.62	p=0.02
Sex			
Female	1		
Male	1.90	1.10-3.29	p=0.022
Ethnic			
White	1		
Other races	6.7×10^{-5}	4.4×10^{-5}-1.02×10^{-4}	p<0.001
College or university degree			
No	1		
Yes	0.94	0.56-1.58	p=0.82
BMI, kg/m^2			
<30	1		
≥30	1.11	0.67-1.83	p=0.69
Waist circumference, cm			
<90	1		
≥90	0.87	0.48-1.57	p=0.64
Number of times per week consuming processed meats			
None	1		
1~3	1.14	0.46-2.83	p=0.78
4+	0.68	0.16-2.94	p=0.61
Number of times per day consuming fruit or vegetables			
None	1		
1~2	0.61	0.27-1.33	p=0.21
3~4	0.51	0.23-1.14	p=0.10
5+	0.39	0.13-1.15	p=0.088
Alcohol consumption			
Daily or almost daily	1		
Three or four times a week	0.56	0.29-1.10	p=0.093
Once or twice a week	0.72	0.39-1.33	p=0.30
One to three times a month	1.04	0.54-2.01	p=0.91
Special occasions only	0.60	0.26-1.36	p=0.22
Never	1.16	0.55-2.45	p=0.70
Smoking history			
No	1		
Yes	2.06	1.21-3.49	p=0.008
Days per week spent doing moderate physical activity >10 mins			
None	1		
1~2	1.07	0.54-2.12	p=0.85
3~7	0.87	0.48-1.58	p=0.65

LDLC, mmol/L			
Hypercholesterolaemia	1		
≥ 4.1	1.57	0.66-3.77	p=0.31
≥3.4<4.1	1.70	0.75-3.86	p=0.21
≥2.6<3.4	1.03	0.46-2.32	p=0.94
<2.6	1.92	0.97-3.77	p=0.059
HDLC, mmol/L			
<1	1		
≥1	0.53	0.31-0.89	p=0.016
TG, mmol/L			
≥2.3	1		
≥1.7<2.3	0.97	0.54-1.75	p=0.93
<1.7	1.14	0.69-1.90	p=0.61
Family history of cardiovascular disease			
No	1		
Yes	0.87	0.56-1.34	p=0.53
Hypertension			
No	1		
Yes	2.21	1.19-4.08	p=0.012
Diabetes			
No	1		
Yes	1.56	0.71-3.41	p=0.27
Coronary artery disease			
No	1		
Yes	1.83	0.99-3.39	p=0.054
Heart failure			
No	1		
Yes	6.02	0.79-45.7	p=0.083
Atrial fibrillation			
No	1		
Yes	2.94	1.21-7.15	p=0.017
Ischaemic stroke			
No	1		
Yes	1.28	0.52-3.17	p=0.59
On antihypertensive medication			
Nonuser	1		
User	1.12	0.62-2.01	p=0.71
On statin			
Nonuser	1		
User	1.36	0.67-2.73	p=0.40
On diabetes medication			
Nonuser	1		
User	1.11	0.45-2.72	p=0.83
Chemotherapy			

No	1		
Yes	1.66	0.95-2.90	p=0.074
Radiotherapy			
No	1		
Yes	2.15	0.89-5.17	p=0.089
CRP, mg/L			
≤2	1		
>2	1.83	1.15-2.89	p=0.01

Table S7. Associations Between CRP and Cardiovascular Mortality in Cancer Survivors for Model 3 when CRP was Categorised by Specified Clinical Cut Points

	sHR	95%CI	P value
Age, years			
<65	1		
≥65	1.69	1.12-2.56	p=0.013
Sex			
Female	1		
Male	1.94	1.16-3.26	p=0.012
Ethnic			
White	1		
Other races	0.38	0.05-2.75	p=0.34
College or university degree			
No	1		
Yes	0.90	0.55-1.46	p=0.67
BMI, kg/m^2			
<30	1		
≥30	1.05	0.65-1.70	p=0.84
Waist circumference, cm			
<90	1		
≥90	0.90	0.51-1.57	p=0.71
Number of times per week consuming processed meats			
None	1		
1~3	1.30	0.52-3.27	p=0.58
4+	0.65	0.15-2.78	p=0.56
Number of times per day consuming fruit or vegetables			
None	1		
1~2	0.72	0.33-1.58	p=0.41
3~4	0.56	0.25-1.23	p=0.15
5+	0.45	0.16-1.27	p=0.13
Alcohol consumption			
Daily or almost daily	1		
Three or four times a week	0.58	0.31-1.08	p=0.088
Once or twice a week	0.64	0.35-1.15	p=0.13
One to three times a month	1.07	0.58-2.00	p=0.82
Special occasions only	0.58	0.27-1.26	p=0.17
Never	1.25	0.64-2.45	p=0.52
Smoking history			
No	1		
Yes	1.92	1.17-3.14	p=0.01
Days per week spent doing moderate physical activity >10 mins			
None	1		
1~2	1.05	0.54-2.01	p=0.89
3~7	0.85	0.48-1.48	p=0.56

LDLC, mmol/L			
Hypercholesterolaemia	1		
≥ 4.1	1.62	0.71-3.68	p=0.25
≥3.4<4.1	1.55	0.72-3.32	p=0.26
≥2.6<3.4	0.96	0.46-2.02	p=0.92
<2.6	1.78	0.94-3.35	p=0.074
HDLC, mmol/L			
<1	1		
≥1	0.53	0.32-0.87	p=0.012
TG, mmol/L			
≥2.3	1		
≥1.7<2.3	0.92	0.54-1.59	p=0.77
<1.7	0.97	0.60-1.56	p=0.89
Family history of cardiovascular disease			
No	1		
Yes	0.88	0.58-1.33	p=0.54
Hypertension			
No	1		
Yes	2.14	1.23-3.74	p=0.007
Diabetes			
No	1		
Yes	1.46	0.70-3.06	p=0.31
Coronary artery disease			
No	1		
Yes	2.06	1.19-3.59	p=0.01
Heart failure			
No	1		
Yes	5.56	0.73-42.5	p=0.10
Atrial fibrillation			
No	1		
Yes	3.14	1.37-7.19	p=0.007
Ischaemic stroke			
No	1		
Yes	1.28	0.55-2.97	p=0.57
On antihypertensive medication			
Nonuser	1		
User	1.06	0.62-1.83	p=0.83
On statin			
Nonuser	1		
User	1.41	0.74-2.67	p=0.30
On diabetes medication			
Nonuser	1		
User	1.09	0.46-2.56	p=0.85
Chemotherapy			

No	1		
Yes	1.58	0.94-2.66	p=0.084
Radiotherapy			
No	1		
Yes	2.08	0.92-4.72	p=0.08
CRP, mg/L			
≤1	1		
>1<3	1.56	0.85-2.87	p=0.15
≥3	1.99	1.09-3.66	p=0.026

Table S8. Associations Between CRP and Cardiovascular Mortality in Cancer Survivors for Model 3 when CRP was Categorised by Quartile

	sHR	95%CI	P value
Age, years			
<65	1		
≥65	1.68	1.11-2.54	p=0.014
Sex			
Female	1		
Male	1.98	1.18-3.32	p=0.01
Ethnic			
White	1		
Other races	0.39	0.05-2.79	p=0.35
College or university degree			
No	1		
Yes	0.92	0.56-1.49	p=0.72
BMI, kg/m^2			
<30	1		
≥30	1.04	0.65-1.67	p=0.86
Waist circumference, cm			
<90	1		
≥90	0.87	0.50-1.51	p=0.62
Number of times per week consuming processed meats			
None	1		
1~3	1.28	0.51-3.20	p=0.60
4+	0.63	0.15-2.72	p=0.54
Number of times per day consuming fruit or vegetables			
None	1		
1~2	0.72	0.33-1.57	p=0.41
3~4	0.56	0.25-1.23	p=0.15
5+	0.45	0.16-1.27	p=0.13
Alcohol consumption			
Daily or almost daily	1		
Three or four times a week	0.57	0.31-1.08	p=0.085
Once or twice a week	0.63	0.35-1.14	p=0.13
One to three times a month	1.06	0.57-1.97	p=0.86
Special occasions only	0.58	0.27-1.25	p=0.16
Never	1.24	0.64-2.43	p=0.53
Smoking history			
No	1		
Yes	1.91	1.16-3.12	p=0.01
Days per week spent doing moderate physical activity >10 mins			
None	1		
1~2	1.06	0.55-2.03	p=0.86
3~7	0.85	0.49-1.49	p=0.58

LDLC, mmol/L			
Hypercholesterolaemia	1		
≥ 4.1	1.62	0.71-3.71	p=0.26
≥3.4<4.1	1.56	0.72-3.36	p=0.26
≥2.6<3.4	0.97	0.46-2.05	p=0.93
<2.6	1.81	0.96-3.41	p=0.069
HDLC, mmol/L			
<1	1		
≥1	0.53	0.32-0.87	p=0.012
TG, mmol/L			
≥2.3	1		
≥1.7<2.3	0.92	0.53-1.59	p=0.76
<1.7	0.98	0.60-1.58	p=0.92
Family history of cardiovascular disease			
No	1		
Yes	0.88	0.58-1.33	p=0.55
Hypertension			
No	1		
Yes	2.12	1.21-3.69	p=0.008
Diabetes			
No	1		
Yes	1.47	0.70-3.09	p=0.31
Coronary artery disease			
No	1		
Yes	2.06	1.18-3.59	p=0.011
Heart failure			
No	1		
Yes	5.49	0.72-42.1	p=0.1
Atrial fibrillation			
No	1		
Yes	3.12	1.35-7.19	p=0.008
Ischaemic stroke			
No	1		
Yes	1.28	0.55-2.99	p=0.56
On antihypertensive medication			
Nonuser	1		
User	1.07	0.62-1.85	p=0.80
On statin			
Nonuser	1		
User	1.39	0.73-2.65	p=0.31
On diabetes medication			
Nonuser	1		
User	1.08	0.45-2.57	p=0.86
Chemotherapy			

No	1		
Yes	1.57	0.93-2.63	p=0.089
Radiotherapy			
No	1		
Yes	2.07	0.91-4.70	p=0.082
CRP, mg/L			
≤0.9	1		
>0.9<2	2.10	0.99-4.45	p=0.054
≥2<3.8	2.37	1.14-4.90	p=0.021
≥3.8	2.85	1.40-5.83	p=0.004

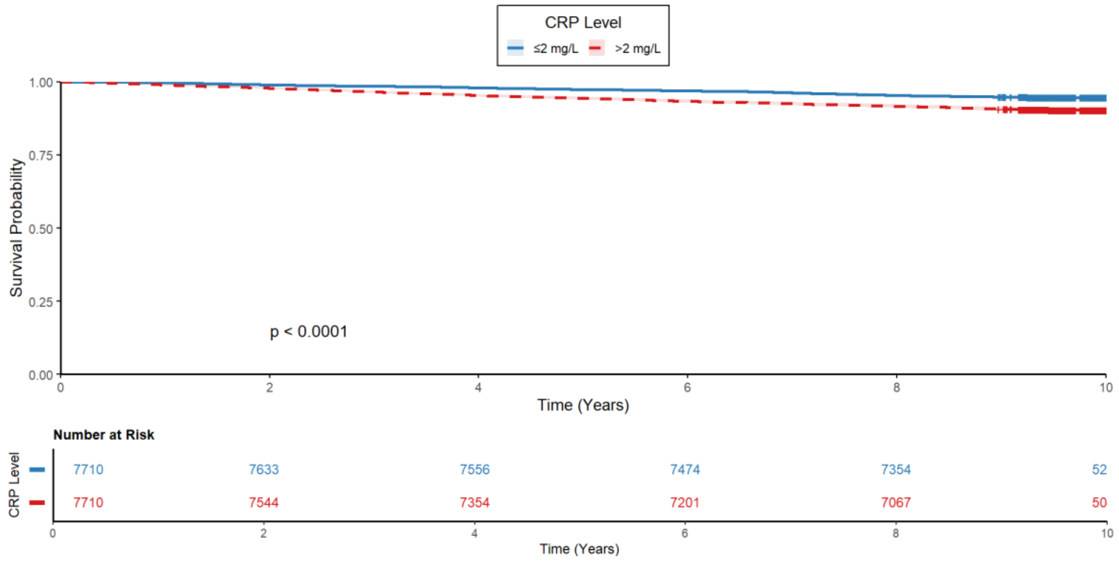

FigureS1: Associations between CRP levels and All Cause Mortality in Cancer Survivors. A univariate analysis was performed, plotting Kaplan-Meier survival curves for cancer patients, and comparing group differences using the Log-rank test.

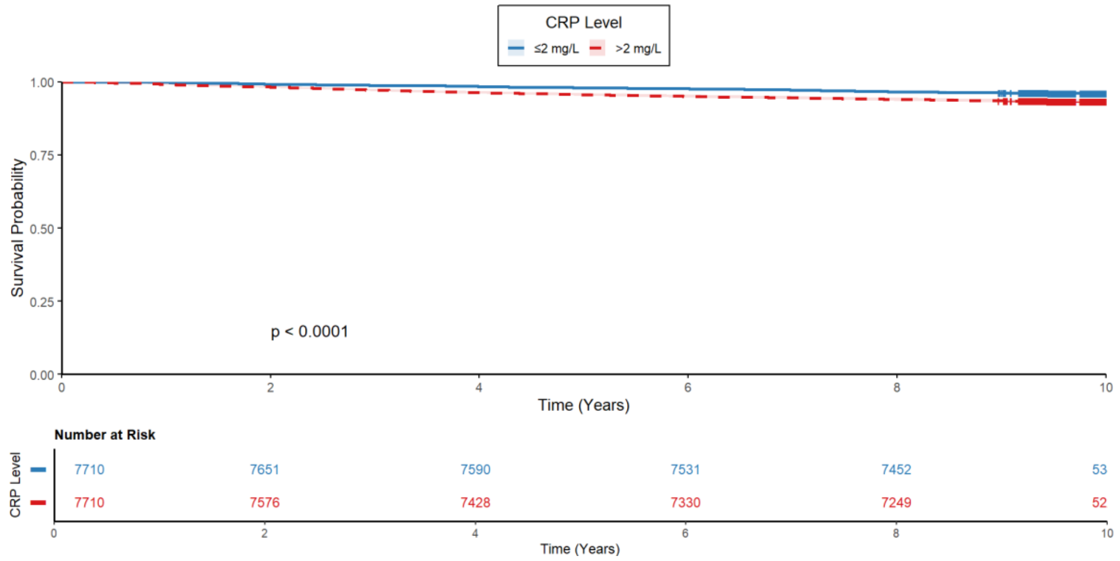

FigureS2: Associations between CRP levels and Cancer Mortality in Cancer Survivors. A univariate analysis was performed, plotting Kaplan-Meier survival curves for cancer patients, and comparing group differences using the Log-rank test.
